# Vitamin D decreases tumor cell growth and invasiveness in primary malignant bone tumors in vitro

**DOI:** 10.1016/j.jbo.2026.100792

**Published:** 2026-07-31

**Authors:** M. Hein, S. Zeck, M. Krug, J. Meißner-Weigl, O. Bahr, K. Kriukov, M. Kuric, D. Docheva, M. Rudert, K. Horas, R. Ebert

**Affiliations:** a University of Würzburg, Department of Musculoskeletal Tissue Regeneration, Orthopedic Hospital König-Ludwig-Haus, Friedrich-Bergius-Ring 15, 97076 Würzburg, Germany; b University of Würzburg, Department of Orthopedics, Orthopedic Hospital König-Ludwig-Haus, Brettreichstrasse 11, 97074 Würzburg, Germany; c ATOS Klinik Wiesbaden, Hagenauer Straße 47, 65203 Wiesbaden, Germany

**Keywords:** Primary malignant bone tumors, Osteosarcoma, Chondrosarcoma, Ewing's sarcoma, 1,25(OH)2 vitamin D3

## Abstract

Primary malignant bone tumors (PMBTs) are rare neoplasms arising from bone tissue. Limited evidence suggests an association between reduced serum 25(OH) vitamin D3 levels and PMBT incidence. The active metabolite 1,25(OH)2 vitamin D3 (1,25D3) modulates tumor cell proliferation, apoptosis, and migration through interactions with the vitamin D receptor (VDR).

This study investigated the effects of 1,25D3 on osteosarcoma (MG-63, SaOS-2), chondrosarcoma (SW-1353), and Ewing's sarcoma (RD-ES) cell lines. Expression of key components of vitamin D metabolism, including 1α-hydroxylase (CYP27B1), 24-hydroxylase (CYP24A1), VDR, protein disulfide-isomerase A3 (PDIA3), and the 1,25D3-responsive bone gamma-carboxyglutamate protein (BGLAP), was analyzed at mRNA and protein levels. VDR nuclear translocation was assessed by Western blotting. Cell viability, cell cycle progression, apoptosis, and migration were evaluated using metabolic assays, flow cytometry, immunocytochemistry, wound healing, and live-cell imaging.

Treatment with 1,25D3 upregulated CYP24A1 in osteosarcoma cells, while BGLAP expression was detected in both osteosarcoma and SW-1353 cells. All examined cell lines expressed VDR and PDIA3, and 1,25D3 induced VDR nuclear translocation. MG-63 cell viability decreased by 23%, whereas SW-1353 cell viability decreased by 10%, with no significant changes observed in SaOS-2 and RD-ES cells. In MG-63 cells, 1,25D3 induced a significant accumulation of cells in the G1 phase, consistent with cell-cycle arrest, whereas only minor cell-cycle alterations were observed in the other cell lines. Interestingly, 1,25D3 reduced the proportion of apoptotic osteosarcoma cells by 18–30%. The migration of MG-63 cells was significantly reduced by 1,25D3, and an inhibitory trend, albeit not statistically significant, was also observed in RD-ES cells.

In conclusion, 1,25D3 modulated cell viability, apoptosis, and migration in PMBT cell models, with distinct, cell-type-specific responses. These findings suggest a potential role for 1,25D3 in regulating PMBT growth and invasiveness.

## Highlights

• Osteosarcoma cells express 1,25D3 responsive genes.
• Chondrosarcoma and Ewing's sarcoma cells partially express 1,25D3 responsive genes.
• 1,25D3 reduces osteosarcoma and chondrosarcoma cell viability.
• 1,25D3 decreases MG-63 cell migration.
• 1,25D3 has a potential role in suppressing PMBT growth and invasiveness.

## Graphical abstract

**Figure:**
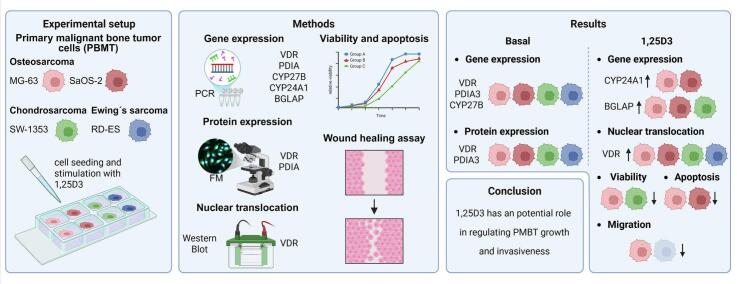

## 1 Introduction

Primary malignant bone tumors (PMBTs), a rare group of neoplasms originating from bone tissue, including osteosarcoma, chondrosarcoma, and Ewing's sarcoma as the predominant subtypes. Osteosarcoma, the most common type of PMBTs, is characterized by osteoid formation. The tumor typically arises in the metaphyses of long bones during adolescence and shows high metastatic potential [1], [2]. The less frequent chondrosarcoma exhibits slower local progression, a metastatic risk highly linked to tumor differentiation, and mainly occurs in adults in regions such as the pelvis, femur, or ribs [3]. Ewing's sarcoma, an aggressive tumor predominantly affecting children and adolescents, is driven by a characteristic EWS-FLI1 gene translocation and arises most commonly in the femur and pelvis [4]. Despite their clinical and pathological heterogeneity, tumors share common challenges in early diagnosis due to nonspecific symptoms. The prognosis of patients affected by PMBTs remains poor, with survival rates ranging from 50 to 70%. Young patients are typically confronted with radical surgical interventions and aggressive chemotherapy [5], [6]. Given these challenges, the identification of new therapeutic and preventive strategies is highly clinically relevant.

In addition to its classical roles in calcium homeostasis and bone health, vitamin D exerts anticancer effects through its active form, 1,25-dihydroxyvitamin D3 (1,25D3), which mediates cellular proliferation, apoptosis, differentiation, and migration via the vitamin D receptor (VDR) [7]. VDR, a nuclear receptor activated by 1,25D3, regulates the transcription of genes such as the catabolic enzyme 24-hydroxylase (CYP24A1), forming a feedback loop that limits hormone activity [8]. In addition to this classical VDR-dependent pathway, recent work has identified a non-canonical vitamin D metabolic route initiated by the cholesterol side-chain cleavage enzyme (CYP11A1) [9]. This enzyme generates a series of hydroxyvitamin D metabolites that exert biological activity through alternative nuclear receptors, including retinoic acid receptor related orphan receptor (ROR)α and RORγ and the aryl hydrocarbon receptor (AhR), expanding the spectrum of vitamin D-mediated signaling [10], [11]. These pathways have been implicated in antiproliferative and anti-inflammatory responses and may contribute to tumor-specific vitamin D effects independent of 1,25D3.

Notably, aberrant expression of vitamin D metabolic enzymes, including 1α-hydroxylase (CYP27B1) and CYP24A1, has been observed in various malignancies, suggesting their role in modulating the anticancer effects of 1,25D3 [12], [13]. Elevated CYP24A1 levels, for example, have been associated with reduced 1,25D3 efficacy in prostate, colorectal, and breast cancers [14], [15]. Furthermore, decreased VDR expression has been linked to increased metastatic potential, as demonstrated in melanoma and breast cancer models [16], [17]. Epidemiological data indicate a correlation between low serum 25-hydroxyvitamin D3 (25D3) levels and increased risk of or progression of PMBTs [18], [19]. Patients with PMBT have significantly lower 25D3 levels compared to those with benign bone tumors or bone metastases, suggesting a potential role of vitamin D deficiency in tumor progression [19]. The precise mechanisms by which vitamin D metabolism and signaling influence PMBTs remain poorly understood, and data specific to these rare tumors are limited.

The selection of cell lines used in this study was based on a deliberate and biologically informed strategy to capture the diversity of PMBTs in the context of vitamin D signaling. Specifically, we included SaOS-2 and MG-63 as well-established human osteosarcoma cell lines that represent canonical models of osteoblastic differentiation and bone-like extracellular matrix production, making them particularly suitable for investigating vitamin D-mediated effects on osteogenic processes [20]. To extend the analysis beyond osteosarcoma, RD-ES (Ewing sarcoma) and SW-1353 (chondrosarcoma) were incorporated as widely used and representative models of their respective tumor entities. RD-ES is a standard Ewing sarcoma model derived from a bone tumor [21], [22], while SW-1353 originates from a primary chondrosarcoma and is among the most frequently used chondrosarcoma cell lines [21], [23]. This approach enables the assessment of VDR signaling across the three most common types of PMBTs. Although numerous additional cell lines are available, our selected panel provides a balanced and widely accepted representation of bone-derived sarcoma entities. While VDR expression has been reported in various sarcoma subtypes [24], its interaction with the distinct molecular backgrounds of Ewing sarcoma and chondrosarcoma remains less well characterized than in osteosarcoma. Thus, inclusion of these models allows us to explore whether vitamin D responsiveness is conserved across biologically diverse sarcoma entities.

For this reason, this study aimed to investigate the responsiveness of osteosarcoma, chondrosarcoma, and Ewing's sarcoma cell lines to 1,25D3. Specifically, we characterized the expression of key enzymes involved in vitamin D metabolism (CYP27B1, CYP24A1), the VDR, and the alternative vitamin D-binding protein, PDIA3, as well as the impact of 1,25D3 on gene expression, cell cycle, cell viability, apoptosis, and migration. Therefore, we wanted to determine the prophylactic or therapeutic potential of vitamin D in PMBT and identify tumor-specific patterns of responsiveness.

## 2 Materials and methods

### 2.1 Cell culture

Human MG-63 osteosarcoma (RRID:CVCL_0426) and human SW-1353 chondrosarcoma cells (RRID:CVCL_0543) were cultivated in DMEM/F-12 with GlutaMAX™ supplemented with 5% (*v*/v) FCS (Bio&Sell GmbH, Feucht, Germany), 100 U/mL penicillin, and 100 μg/mL streptomycin. Human SaOS-2 osteosarcoma cells (RRID:CVCL_0548) were cultivated in McCoy's 5 A medium containing GlutaMAX™ supplemented with 5% (*v*/v) FCS, 100 U/mL penicillin, and 100 μg/mL streptomycin. Human RD-ES Ewing's sarcoma cells (RRID:CVCL_2169) were cultivated in RPMI 1640 medium supplemented with 10% FCS, 1% l-glutamine, and 2.5 g/L glucose (Sigma Aldrich GmbH, Schnelldorf, Germany). The MG-63, SW-1353, and RD-ES cell lines were obtained from CLS Cell Lines Service GmbH (Eppelheim, Germany), and the SaOS-2 cell line was acquired from the Leibniz Institute DSMZ GmbH (Braunschweig, Germany). Media and additives were purchased from Thermo Fisher Scientific GmbH (Darmstadt, Germany) unless otherwise stated. The cells were grown at 37 °C in a humidified atmosphere containing 5% CO_2_.

### 2.2 Cell viability and apoptosis assays

To examine cell viability and apoptosis, the cells were seeded in 96-well plates (1 × 10^3^ cells per well), allowed to adhere at 37 °C and 5% CO_2_ overnight, and then stimulated with 10^−7^ M 1,25D3 for 48 h and 0.01% ethanol (EtOH) as the solvent control. Viability and apoptosis rates were assessed via the CellTiter-Glo® Luminescent Cell Viability Assay and the Caspase-Glo® 3/7 Assay, respectively (both from Promega GmbH, Mannheim, Germany), according to the manufacturer's instructions. Luminescence was measured with an Orion II Luminometer (Berthold Detection Systems, Pforzheim, Germany). The data are expressed as the mean ± standard error of the mean (SEM) from four independent experiments, each with technical duplicates or triplicates.

### 2.3 Cell cycle analysis

Prior to cell cycle analysis MG-63 and SaOS-2 osteosarcoma, SW1353 chondrosarcoma, and RD-ES Ewing sarcoma cells were subjected to serum starvation to synchronize cell cycle progression. Briefly, 1 × 10^4^ cells/cm^2^ were cultured in medium containing 2% FCS for 72 h before treating with 1,25D3 for 24 h and 48 h. Afterwards, cells were collected by centrifugation at 1000 x*g* for 5 min at 4 °C. The pellet was washed with 5 mL of ice-cold PBS, centrifuged, and resuspended in 500 μL PBS, ensuring a single-cell suspension. 4.5 mL of ice-cold 70% ethanol was slowly added for fixation, with gentle vortexing. Fixed samples were stored at 4 °C for short-term use.

On the day of analysis, ethanol-fixed cells were centrifuged at 1000 x*g* for 5 min and resuspended in 5 mL PBS and incubated for 15 min at room temperature to allow rehydration. After centrifugation at 1000 x*g* for 5 min, cell pellets were resuspended in 500 μL staining solution (10 μg/mL DAPI / 0.1% Triton X-100 in PBS) and incubated for 30 min at room temperature, protected from light. Samples were transferred to appropriate cytometry tubes and analyzed by flow cytometry. DAPI fluorescence was detected using excitation at 405 nm and emission collected with a 450/50 nm filter. Data of three independent experiments were acquired in linear mode at a low flow rate with a BDTM LSR II X device (BD Biosciences, Heidelberg, Germany) and analyzed using the free online software Floreada.io (https://floreada.io).

### 2.4 RNA isolation and polymerase chain reaction

The cells (*n* = 4) were treated with 10^−7^ M 1,25D3 or 0.01% EtOH as a solvent control for 24 h, and total RNA was isolated via the NucleoSpin® RNA II Purification Kit (Macherey-Nagel, Düren, Germany) according to the manufacturer's instructions. For cDNA synthesis, 1 μg of total RNA was reverse transcribed with Oligo(dT)15 primers (1 μL, 25 pmol) and 1 μL of M-MLV Reverse Transcriptase RNase (H-) point mutant (200 U/μL), 5× reaction buffer (5 μL), nuclease-free H_2_O (0.37 μL), and dNTPs (0.67 μL) (20 mM dATP, dTTP, dGTP, or dCTP) in a volume of 25 μL at 42 °C for 60 min, followed by a denaturation step at 95 °C for 10 min. All reagents were purchased from Promega GmbH. The cDNA was diluted 1:2 with nuclease-free H_2_O and stored at −20 °C.

For RT-PCR, 1 μL of cDNA was used as a template in a volume of 50 μL. The PCR conditions were as follows: GoTaq DNA polymerase (0.2 μL), 5× PCR buffer (10 μL), dNTPs (0.5 μL, 20 mM each), forward primer (0.5 μL, 10 pmol/μL), reverse primer (0.5 μL, 10 pmol/μL), MgCl_2_, and nuclease-free H_2_O (adjusted to a total volume of 48 μL). The diluted cDNA (2 μL) was then added to the PCR mixture. All reagents, except for the primers, were purchased from Promega GmbH. Details of the primers, including their sequence, MgCl_2_ concentration, annealing temperature, and product size, are listed in Table 1. RPS27A was used as a housekeeping gene, and the resulting PCR bands were analyzed via agarose gel electrophoresis and band intensities were quantified with ImageJ.

**Table 1:** PCR primers and conditions. The primer names, sequences, product lengths, annealing temperatures and MgCl_2_ concentrations are shown. CYP24A1 primers amplify two transcript variants (transcript variants 1 and 5).

Primer	Sequence (5′ - 3′)	product length (bp)	annealing temp (°C)	MgCl_2_ (mM)
CYP24A1_for	TATGAGGCTTACGCCGAGTG	445 617	59	2
CYP24A1_rev	CCGCTTCCCTGAGTTGGATA			
CYP27B1_for	ACGCTCTCTTGGGCTCTGTA	203	56	2
CYP27B1_rev	TCTGGGACACGAGAATTTCC			
BGLAP_for	ATGAGAGCCCTCACACTCCTC	293	61	1.5
BGLAP_rev	GCCGTAGAAGCGCCGATAGGC			
PDIA3_for	TGCTAGAACTCACGGACGAC	260	61	1
PDIA3_rev	CACCTGCTTCTTCACCATCTC			
RPS27A_for	TCGTGGTGGTGCTAAGAAAA	141	59	2
RPS27A_rev	TCTCGACGAAGGCGACTAAT			
VDR_for	TGACCCTGGAGACTTTGACC	498	55	2
VDR_rev	GCTGGGAGTGTGTCTGGAGT			

### 2.5 Immunocytochemistry

A total of 2 × 10^4^ cells were seeded on cover slips in 12-well plates, and after 24 h, they were treated with 10^−7^ M 1,25D3 or 0.01% EtOH as a solvent control for 2 h, washed twice with PBS, and fixed for 10 min with −20 °C cold methanol. The cells were washed thrice for 5 min with PBS, permeabilized with 0.1% Triton-X-100/PBS, washed again with PBS, and blocked for VDR staining with 1% BSA/22.52 mg/mL glycine/0.1% Tween in PBS or for PDIA3 staining with 4% FCS/PBS, each for 30 min. The cells were incubated with primary antibodies against PDIA3 (anti-PDIA3 antibody produced in rabbit, 1:200, HPA002645, Sigma Aldrich GmbH) in blocking solution and against VDR (anti-VDR (C-20) antibody, produced in rabbit, 1:250, sc-1008, Santa Cruz Biotechnology, Heidelberg, Germany) in 1% BSA/PBS overnight at 4 °C. The cells were washed thrice with PBS for 5 min and incubated with Alexa Fluor 488-conjugated secondary antibody (goat anti-rabbit IgG, 1:800, A11008, Thermo Fisher Scientific) in 1% BSA/PBS or 4% FCS for 1.5 h in the dark and washed thrice with PBS for 5 min. The coverslips were transferred to slides with a drop of Vectashield Mounting Medium with DAPI (Biozol, Eching, Germany) and analyzed with a ZEISS Axio Observer 7 fluorescence microscope equipped with an Apotome and an Axiocam 506 mono (Carl Zeiss AG, Oberkochen, Germany) with a 20× objective. The same exposure time settings and calibration algorithms were used for all comparable samples. Representative images of two to three independent experiments are shown.

### 2.6 Nuclear extract preparation and Western blot

Nuclear extracts were prepared from cultured cells (*n* = 6), and all steps were conducted on ice or at 4 °C to preserve protein integrity. The cells grown in 25 cm^2^ flasks were washed twice with cold PBS before 1 mL of lysis buffer was added (100 mM HEPES, pH 7.8; 10 mM KCl; 2 mM MgCl₂; 1 mM DTT; and cOmplete™ protease inhibitor, all from Sigma Aldrich). The cells were incubated on ice for 15 min, detached by scraping, and transferred to microcentrifuge tubes. To the lysate, 25 μL of 10% Nonidet P-40 was added, and the mixture was mixed gently, followed by centrifugation at 13,000 ×*g* for 30 s at 4 °C to pellet the nuclei. The nuclear pellet was carefully resuspended in 50 μL of extraction buffer (50 mM HEPES, pH 7.8; 50 mM KCl; 300 mM NaCl; 1 mM DTT; 0.1 mM EDTA; 10% glycerol; and cOmplete™ protease inhibitor) via a trimmed pipette tip to minimize shear forces. After incubation on ice for 20 min, the samples were snap-frozen in liquid nitrogen and stored at −80 °C.

Proteins were quantified via the Pierce BCA protein assay (Thermo Scientific, Darmstadt, Germany) as follows: 15 μg of protein extracts were mixed 1:4 with loading buffer (RotiLoad, Carl Roth GmbH) and denatured by boiling for 5 min. Samples were separated on 12% SDS polyacrylamide gels (375 mM Tris (pH 8.8), 0.1% SDS; 130 mM Tris (pH 6.8), 0.1% SDS). After SDS-PAGE, the proteins were semidry blotted for 90 min at 2.5 mA/cm^2^ at room temperature to an Amersham Protean Supported 0.2 μm nitrocellulose membrane (Merck, Darmstadt, Germany) via a Peqlab HEP-1 Semi-Dry Blotter (VWR, München, Germany). The membranes were blocked with 5% nonfat milk powder in TTBS buffer (0.1 M Tris, 150 mM NaCl, 0.1% Tween-20) and incubated overnight at 4 °C with a primary antibody against the vitamin D3 receptor (D2K6W, rabbit mAB, #12550, 1:1000, Cell Signaling, Leiden, Netherlands), and Lamin B1 (L-5, mouse mAB, #33–2000, 2 μg/mL, Thermo Scientific, Darmstadt, Germany), which were diluted in blocking solution. The membranes were washed 3 × 15 min with TTBS, followed by incubation for 1 h at room temperature with a secondary anti-rabbit IgG-horseradish peroxidase antibody (SH A0545, Sigma Aldrich GmbH) diluted 1:2000, or a secondary anti-mouse IgG- horseradish-peroxidase antibody (#7076S, Cell Signaling, Leiden, Netherlands) diluted 1:1000 in TTBS solution. After another wash for 3 × 15 min, specific staining was detected via a chemiluminescence (ECL) system (VWR München, Germany) according to the manufacturer's instructions. Band intensities were quantified with ImageJ.

### 2.7 Wound healing assay

A total of 3 × 10^4^ cells (*n* = 3) were seeded into each chamber of 2-well silicone culture inserts placed into 8-well chamber slides (both ibidi GmbH, Gräfelfing, Germany) and grown until confluency. The silicone inserts were removed, and the cells were washed with PBS and treated with 10^−6^ M 1,25D3_,_ 10^−7^ M 1,25D3 or 0.01% EtOH as the solvent control. Chamber slides were placed into a live cell incubation chamber (ibidi GmbH), and cell migration and closure of the 500 μm wide gap were monitored by time lapse microscopy (ZEISS Axio Observer 7 microscope equipped with an Axiocam 305 colour camera, where images were acquired every 15 min over 2–3 days). To quantify the area covered by the cells, ZEISS ZEN Intellesis machine learning software was trained to distinguish between the cells and the plastic surface to determine the migration behavior and closure of the gap area. Due to the gap created by the silicone insert, the initial cell-covered area was approximately 60%. The analyses were stopped after gap closure (100% cell coverage). The wound closure rate was calculated from the slope within the linear portion of the area-time curve.

### 2.8 Statistical analysis

All statistical analyses were conducted in GraphPad Prism, version 10.2.2 (GraphPad Software, San Diego, USA).

### 2.9 Graphic software

The graphical abstract was created in BioRender. Ebert, R. (2026) https://BioRender.com/kwnwhn7.

## 3 Results

### 3.1 Distinct gene expression landscapes between PMBT cell lines

**Fig. 1:**
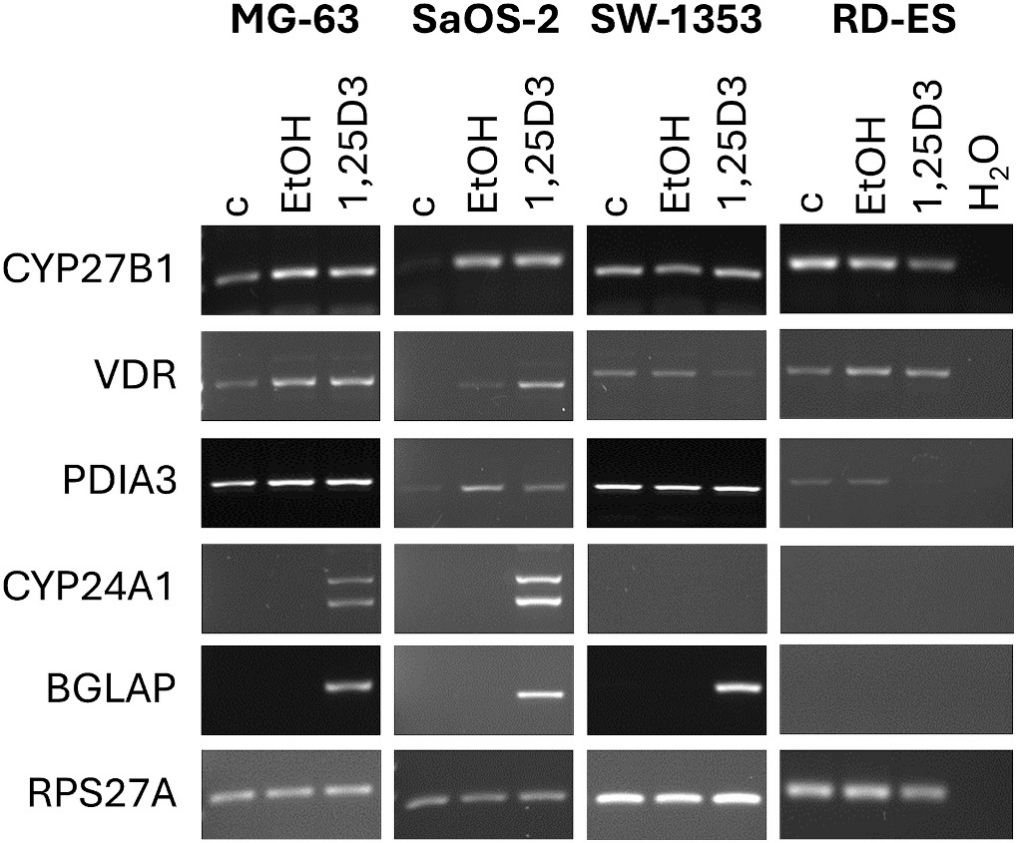
Gene expression analysis. MG-63, SaOS-2, SW-1353, and RD-ES cells were stimulated with 10^−7^ M 1,25D3, with 0.01% ethanol as the solvent control (EtOH), for 24 h. Untreated control samples are labeled (c). The expression of 1α-hydroxylase (CYP27B1), vitamin D receptor (VDR), protein disulfide isomerase A3 (PDIA3), 24-hydroxylase (CYP24A1), bone gamma-carboxyglutamate protein (BGLAP), and ribosomal protein S27a (RPS27A) as the housekeeping gene was analyzed via RT-PCR. One representative analysis of four independent experiments is shown.

An investigation of MG-63, SaOS-2 (both osteosarcoma), SW-1353 (chondrosarcoma) and RD-ES (Ewing's sarcoma) cell lines revealed notable similarities but also differences in gene expression in response to treatment with 10^−7^ M 1,25D3 (Fig. 1; Fig. S2). All four tumor cell lines studied demonstrated consistent basal expression of the genomic vitamin D receptor (VDR) in the absence of 1,25D3 stimulation (Fig. S1A) with no significant differences between cell lines, although MG-63 cells exhibited greater variability compared to SaOS-2, SW-1353, and RD-ES. In addition, all cell lines expressed the membrane-bound vitamin D receptor protein disulfide-isomerase A3 (PDIA3) (Fig. 1; Fig. S2A—D, G), suggesting a possible responsiveness of cells to active vitamin D. The expression of the 25D3-activating enzyme 1α-hydroxylase (CYP27B1) was present throughout all the cell lines, indicating that these cells can synthesize 1,25D3 (Fig. 1; Fig. S2A-E). Notably, the catabolic enzyme 24-hydroxylase (CYP24A1) was detected only after 1,25D3 treatment in the MG-63 and SaOS-2 cell lines. In both lines, two distinct transcript variants were amplified: a 445 bp amplicon corresponding to transcript variant 1, and a 617 bp amplicon corresponding to transcript variant 5 (Fig. 1; Fig. S2A—D, H, I). Ewing's sarcoma and chondrosarcoma cell lines appeared unable to degrade active vitamin D. In addition, we tested the expression of the osteogenic differentiation marker and vitamin D responsive gene bone gamma-carboxyglutamate protein (BGLAP) in response to 1,25D3. Active vitamin D increased the expression of BGLAP in the osteosarcoma (MG-63, SaOS-2) and chondrosarcoma (SW-1353) cell lines, whereas RD-ES did not respond (Fig. 1; Fig. S2A—D, J).

### 3.2 VDR and PDIA3 proteins are expressed in PMBT cell lines

**Fig. 2:**
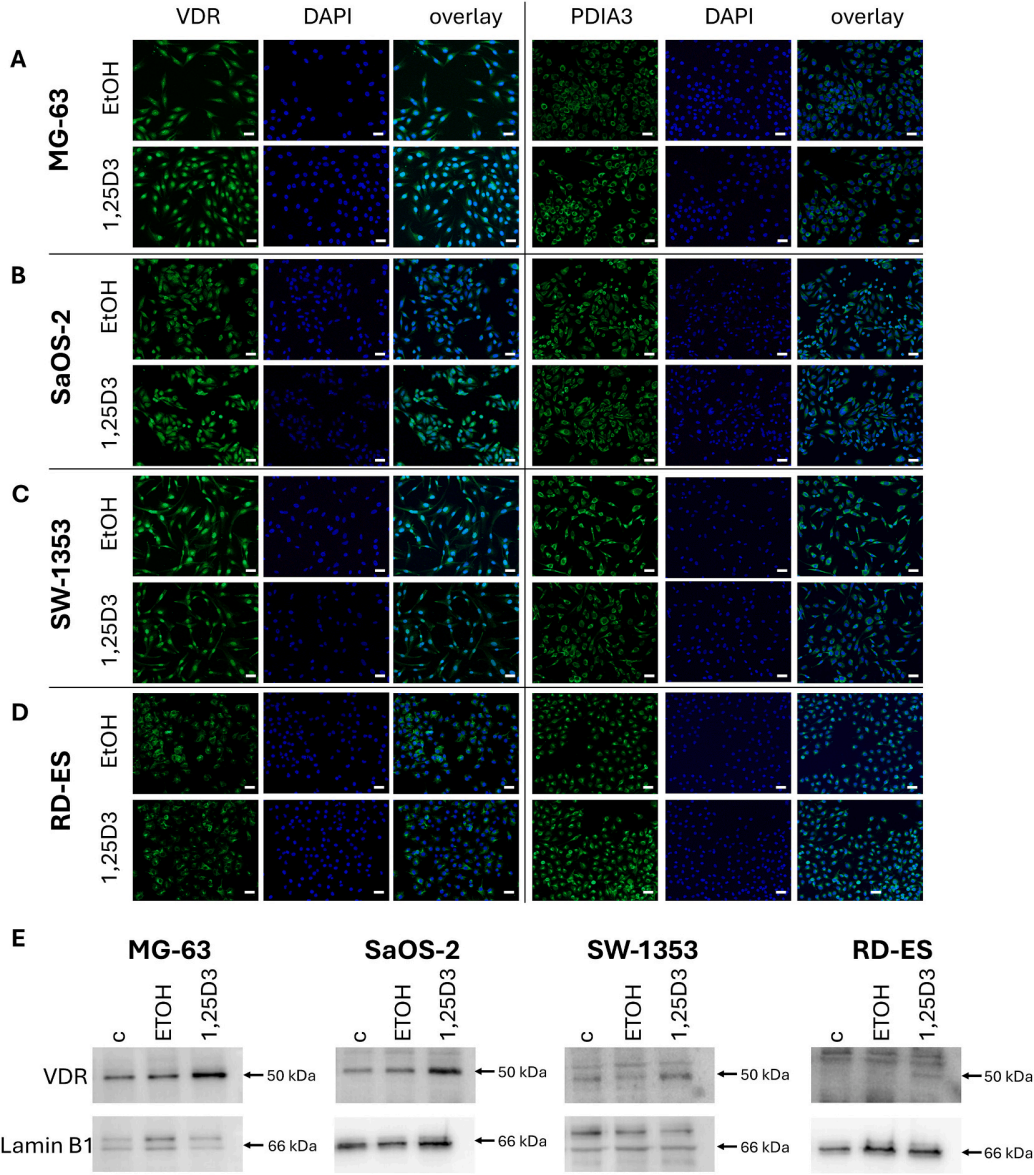
VDR and PDIA3 protein expression in primary malignant bone tumor cell lines. Immunocytochemical detection of nuclear VDR (left) and PDIA3 (right) in MG-63 (A), SaOS-2 (B), SW-1353 (C) and RD-ES (D) cells treated with 10^−7^ M 1,25D3 for 2 h; 0.01% EtOH served as a solvent control. Green staining = Alexa Fluor 488-conjugated secondary antibody, blue staining = DAPI. The bar represents 50 μm. Representative images of two or three independent experiments are shown. (E) Western blot of VDR in the nuclear extracts of MG-63, SaOS-2, SW-1353, and RD-ES cells stimulated with 10^−7^ M 1,25D3 for 2 h. Lamin B1 was stained and served as a loading control. Representative blots of six independent experiments are shown. (For interpretation of the references to colour in this figure legend, the reader is referred to the web version of this article.)

The protein expression of the membrane-bound vitamin D receptor PDIA3 and the nuclear vitamin D receptor VDR was characterized in MG-63 and SaOS-2 osteosarcoma, SW-1353 chondrosarcoma and RD-ES Ewing's sarcoma cell lines through immunocytochemistry (Fig. 2). All four tumor cell lines studied exhibited consistent protein expression of both receptors, confirming the possible responsiveness of cells to active vitamin D. In addition, basal nuclear VDR expression in the absence of 1,25D3 was quantified and compared across all cell lines (Fig. S1B), revealing no significant differences, although MG-63 cells exhibited greater variability than SaOS-2, SW-1353, and RD-ES, consistent with the findings at the mRNA level. After 1,25D3 treatment for 2 h, the osteosarcoma cell lines MG-63 (Fig. 2A, left panel) and SaOS-2 (Fig. 2B, left panel) responded with increased nuclear VDR translocation, but SW-1353 chondrosarcoma (Fig. 2C, left panel) and RD-ES Ewing's sarcoma cells (Fig. 2D, left panel) showed no response. No effect of 1,25D3 on PDIA3 subcellular localization was detected (Fig. 2A-D, right panels). Secondary antibody staining was performed without the use of a primary antibody as a negative control (Fig. S3). VDR translocation was further validated by Western blot analysis of nuclear extracts prepared from MG-63 and SaOS-2 osteosarcoma cells. In contrast to the immunocytochemical staining results, nuclear VDR translocation was also detected in SW-1353 chondrosarcoma and RD-ES Ewing sarcoma cells (Fig. 2E). Densitometric values of the VDR bands (∼55 kDa) were normalized to Lamin B1 (∼66 kDa). Upon treatment with 1,25D3, VDR band intensities increased by 2.3-fold (MG-63), 4.4-fold (SaOS-2), 1.5-fold (SW-1353), and 3.5-fold (RD-ES) after 2 h compared with the 0.01% EtOH solvent control. Numerical data represent means from three independent experiments.

### 3.3 1,25D3 affects the viability and apoptosis of osteosarcoma cell lines

**Fig. 3:**
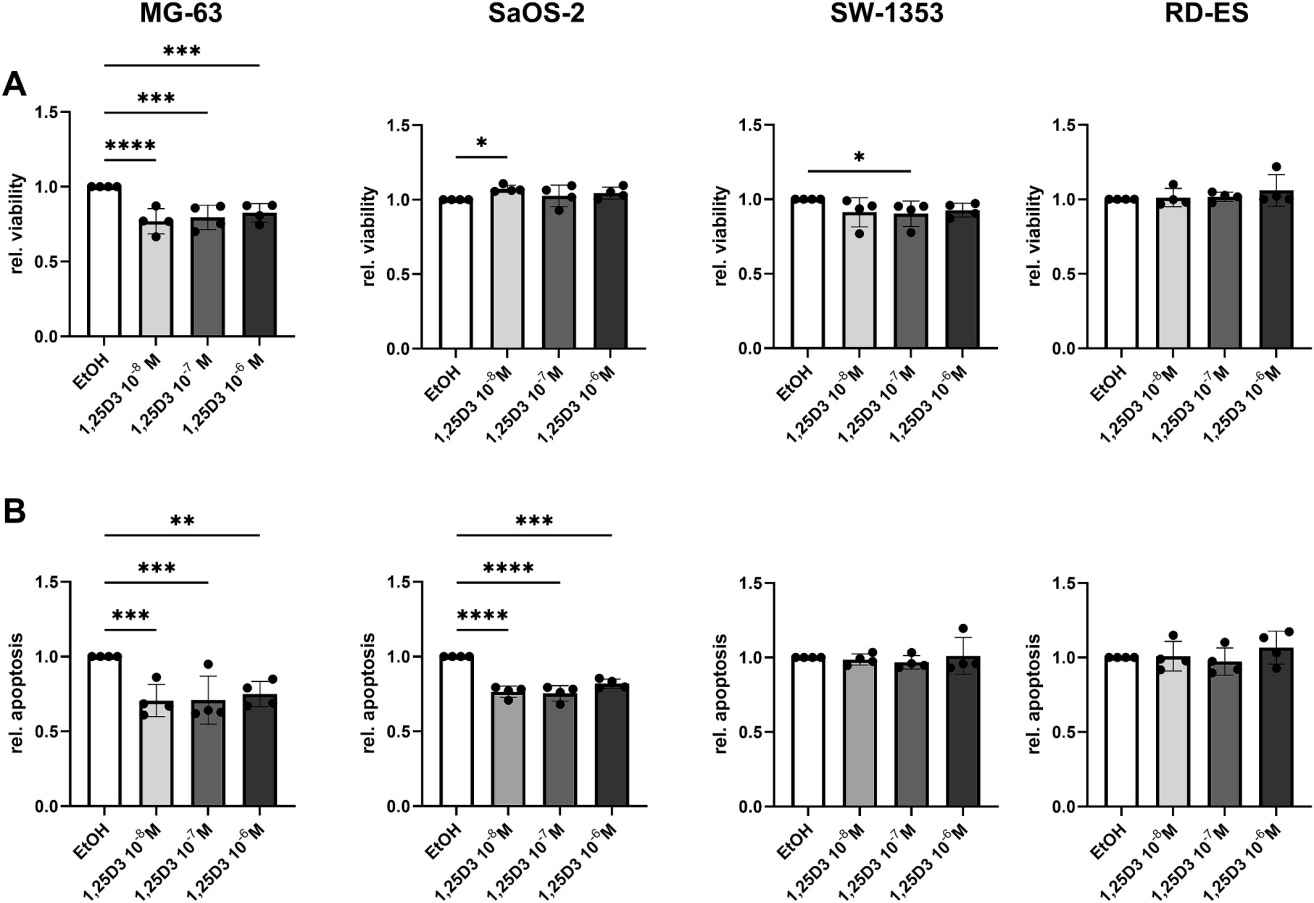
Relative cell viability and apoptosis rates of primary malignant bone tumor cells upon 1,25D3 treatment. MG-63 and SaOS-2 osteosarcoma, SW-1353 chondrosarcoma and RD-ES Ewing sarcoma cells were stimulated with 10^−8^ M, 10^−7^ M, and 10^−6^ M 1,25D3 for 48 h, and the relative viability (A) and apoptosis rates (B) were determined. The data points represent mean values ± SEMs normalized to the solvent (0.01% EtOH) control (*n* = 4), with triplicates in each independent experiment. Statistical analysis was performed via one-way ANOVA in GraphPad Prism (*: *p* < 0.05; **: *p* < 0.01; ***: *p* < 0.001, ****: *p* < 0.0001).

To test the effects on the viability and apoptosis of the MG-63, SaOS-2, SW-1353 and the RD-ES cell lines, 1,25D3 was used at three different concentrations (10^−6^ M, 10^−7^ M_,_ 10^−8^ M). By implementing the Caspase-Glo® 3/7 assay and the CellTiter-Glo® Luminescent Cell Viability assay, significant effects and differences were observed between tumor cell types (Fig. 3). 1,25D3 significantly decreased the viability of MG-63 osteosarcoma cells at all three concentrations used (10^−6^ M, 10^−7^ M_,_ 10^−8^ M). After 48 h of stimulation, the MG-63 cell line showed a maximum reduction of 23% ± 4.2% (10^−8^ M, *p* < 0.0001). The viability of SaOS-2 cells showed a slight but significant increase upon treatment with 10^−8^ M 1,25D3. The viability of SW-1353 (chondrosarcoma) was significantly decreased by 10^−7^ M 1,25D3 (*p* < 0.05), but RD-ES (Ewing's sarcoma) remained unaffected by 1,25D3 (Fig. 3A). Using the Caspase-Glo® 3/7 assay, both osteosarcoma cell lines (MG-63 and SaOS-2) showed a significant decrease in apoptosis at all 1,25D3 concentrations used. Treatment with 10^−7^ M 1,25D3 decreased the percentage of apoptotic cells by 30% ± 8.0% in MG-63 cells (*p* < 0.001) and 25% ± 2.6% in SaOS-2 cells (p < 0.0001). The apoptosis rates of the chondrosarcoma and Ewing's sarcoma cell lines did not change (Fig. 3B).

### 3.4 1,25D3 inhibits the cell cycle of primary malignant bone tumor cells

**Fig. 4:**
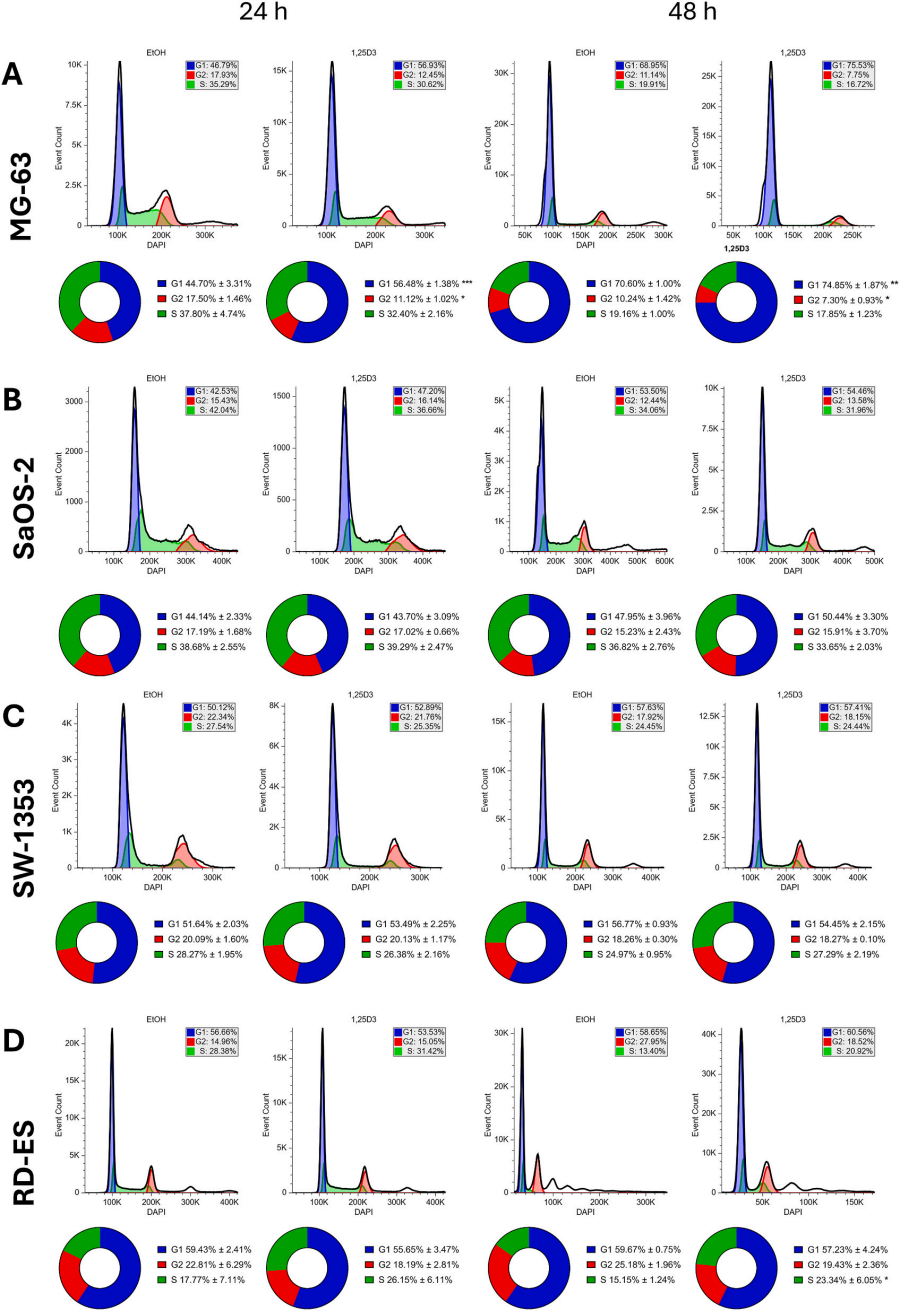
Cell cycle analysis in osteosarcoma, chondrosarcoma, and Ewing's sarcoma cell lines. Cell cycle distribution was analyzed in (A) MG-63 and (B) SaOS-2 osteosarcoma cells, (C) SW-1353 chondrosarcoma cells, and (D) RD-ES Ewing's sarcoma cells. Cells were treated with either 0.01% ethanol solvent control (EtOH) or 10^−7^ M 1,25D3 (1,25D3) for 24 h and 48 h. Graphs show the percentage of cells in the G1 phase (blue), S phase (green), and G2/M phase (red). The line graph represents a representative experiment, with the legend indicating the percentage of cells in each phase. The donut chart shows the mean distribution of cells across the different cell-cycle phases from three independent experiments, presented as mean ± SD. 1,25D3-treated samples were compared with the EtOH solvent control using two-way ANOVA in GraphPad Prism, followed by Šídák's multiple-comparisons test for post hoc pairwise comparisons. Statistical significance is indicated as p < 0.05 (*), p < 0.01 (**), and p < 0.001 (***). (For interpretation of the references to colour in this figure legend, the reader is referred to the web version of this article.)

To investigate the effects of 1,25D3 on cell-cycle progression, MG-63, SaOS-2, SW1353, and RD-ES cells were cultured under 2% serum starvation for 72 h and subsequently stimulated with 10^−7^ M 1,25D3 for 24 h or 48 h. Cell-cycle distribution was analyzed by flow cytometry following DAPI staining. Representative cell-cycle histograms and the corresponding quantitative analyses, presented as donut charts showing the mean values from three independent experiments, are shown in Fig. 4A–D. Detailed statistical results, including mean differences between 1,25D3- and EtOH-treated cells, significance levels, and *p*-values, are provided in Supplementary Table S1.

Among the investigated cell lines, MG-63 cells showed the most marked response to 1,25D3 treatment (Fig. 4A, Supplementary Table S1). After 24 h, the proportion of cells in the G1 phase increased significantly by 11.78%, whereas the G2 fraction decreased significantly by 6.38% compared with EtOH-treated controls. After 48 h, a similar but less pronounced effect was observed, with a significant increase in the G1-phase population of 4.49% and a significant reduction in the G2 fraction of 2.91%. No significant changes were detected in the S-phase population. These results indicate a 1,25D3-induced accumulation of MG-63 cells in the G1 phase, consistent with cell-cycle inhibition.

In contrast, SaOS-2 and SW-1353 cells did not exhibit significant alterations in the distribution of G1, S, or G2 phase cells after either 24 h or 48 h of 1,25D3 treatment (Fig. 4B and C, Supplementary Table S1), indicating that cell-cycle progression in this cell line was largely unaffected by 1,25D3 stimulation.

RD-ES cells showed no significant changes in cell-cycle phase distribution after 24 h of treatment. However, after 48 h, the proportion of S-phase cells increased significantly by 8.19% relative to the control (Fig. 4D, Supplementary Table S1). This increase was accompanied by a decrease in the G2 fraction of 5.75%, although this effect did not reach statistical significance.

Overall, the effects of 1,25D3 on cell-cycle progression were cell line-dependent. MG-63 cells displayed a clear accumulation in the G1 phase accompanied by a reduction in G2 phase cells, indicating cell-cycle arrest. In contrast, SaOS-2 and SW-1353 cells were unresponsive to treatment, whereas RD-ES cells exhibited only modest changes after prolonged exposure to 1,25D3.

### 3.5 1,25D3 decreases migration of primary malignant bone tumor cells

We investigated migration behavior of MG-63 and SaOS-2 (osteosarcoma), SW-1353 (chondrosarcoma), and RD-ES (Ewing's sarcoma) cell lines using live-cell imaging and a wound healing assay. Stimulation with 1,25D3 was performed at two concentrations (10^−7^ M and 10^−6^ M). Basal migration speed differed markedly between cell lines, ranging from approximately 20 h gap closure time in SW-1353 and RD-ES cells to more than 70 h in MG-63 and SaOS-2 cells.

**Fig. 5:**
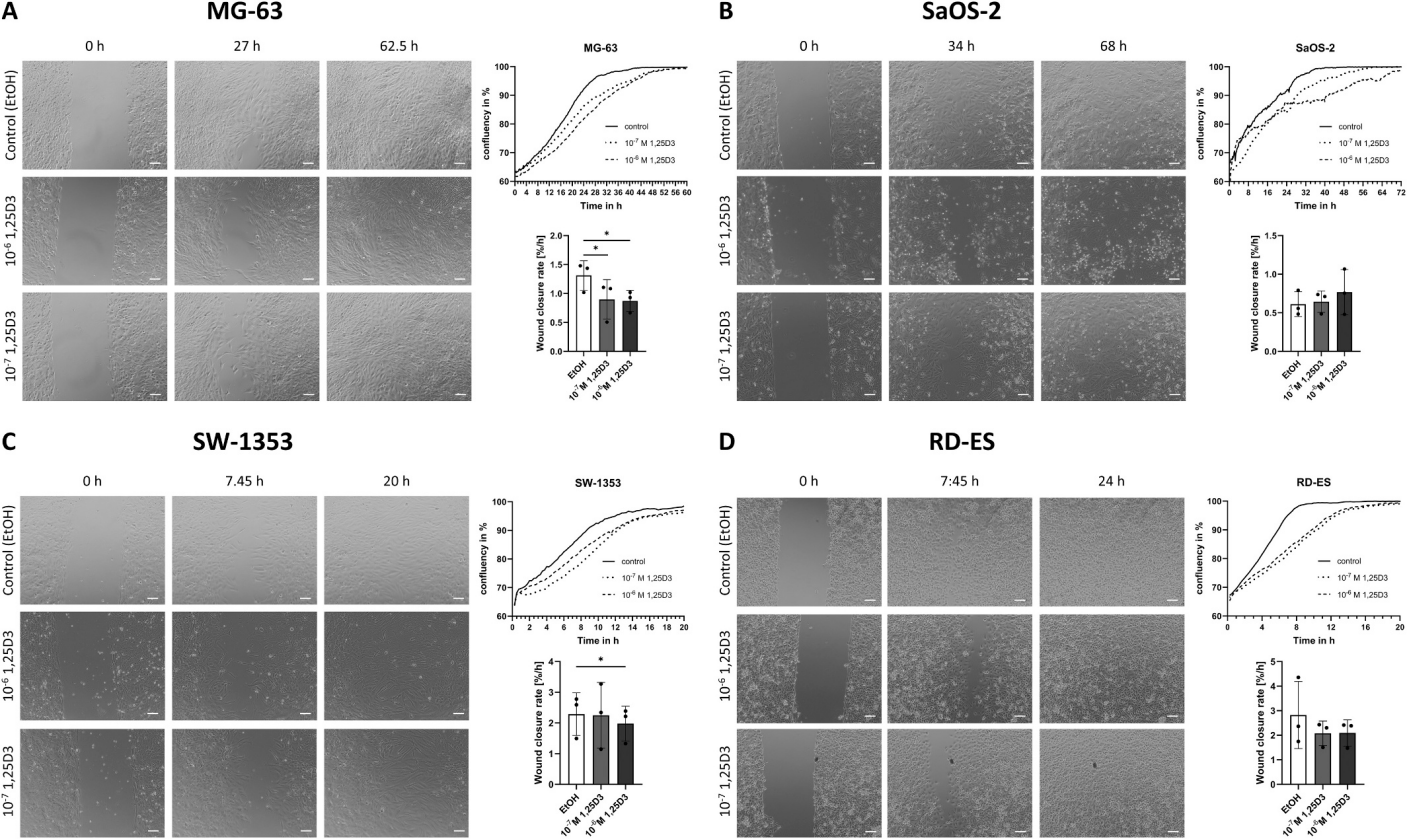
Wound healing assay in osteosarcoma, chondrosarcoma, and Ewing's sarcoma cell lines. Wound healing assay of (A) MG-63 and (B) SaOS-2 osteosarcoma, (C) SW-1353 chondrosarcoma, and (D) RD-ES Ewing's sarcoma cells. Representative images show the 0.01% ethanol solvent control (upper panel), cells treated with 10^−7^ M 1,25D3 (middle panel), and cells treated with 10^−6^ M 1,25D3 (lower panel) at the indicated time points. Scale bar: 100 μm. Line graphs display the quantification of gap closure and cell confluency (%) from a single representative experiment for cells treated with 10^−7^ M 1,25D3 (dotted line), 10^−6^ M 1,25D3 (dashed line), or the 0.01% ethanol control (solid line). Three independent experiments were performed and the bar graph shows the wound closure rate (% per hour) ± SD. Statistical analysis was performed using one-way ANOVA in GraphPad Prism (**: p < 0.01).

Compared with the ethanol solvent control, active vitamin D significantly reduced cell migration in MG-63 osteosarcoma. In MG-63 cells, both 1,25D3 concentrations inhibited migration by more than 30% relative to the solvent control. Representative images and line graphs from one experiment are shown in Fig. 5A, with the most pronounced inhibition visible at 27 h after treatment. However, proliferation-related artefacts affecting MG-63 cell migration cannot be ruled out. In contrast, quantitative and statistical analyses of migration in SaOS-2 osteosarcoma, SW-1353 chondrosarcoma, and RD-ES Ewing's sarcoma cells revealed no statistically significant effects of 1,25D3. Nevertheless, a general trend toward reduced migration was observed in RD-ES cells (Fig. 5D). In addition, individual experiments indicated a similar tendency toward slower migration (Fig. 5B and 5C). Fig. S4 shows line graphs of two further independent experiments for all cell lines.

## 4 Discussion

Our study demonstrates the broad influence of 1,25D3 on cellular processes in osteosarcoma, chondrosarcoma and Ewing's sarcoma cells in vitro. Comparing the expression of relevant receptors and enzymes was the first step in investigating the impact of active vitamin D on PMBT cells. The detection of the two receptors VDR and PDIA3 indicated a possible direct influence of 1,25D3 on tumor cells. A functional confirmation of canonical VDR activation in our models is the strong induction of CYP24A1 (MG-63 and SaOS-2 osteosarcoma cells) and BGLAP (MG-63 and SaOS-2 osteosarcoma, and SW-1353 chondrosarcoma cells) following 1,25D3 treatment. Both genes contain classical vitamin D response elements (VDREs) in their promoter regions and are well-established direct transcriptional targets of ligand-activated VDR. The CYP24A1 promoter harbors two canonical VDREs, and nuclear VDR binding to these elements has been conclusively demonstrated by electrophoretic mobility shift assay (EMSA) [25]. Likewise, the BGLAP (osteocalcin) promoter contains a functional VDRE, with VDR binding validated by EMSA and reporter assays [26]. Consistent with these mechanistic insights, our data show clear nuclear translocation of VDR upon 1,25D3 stimulation, as evidenced by both immunocytochemistry (MG-63; less pronounced in SaOS-2, but clearly detectable in SW-1353) and nuclear fraction Western blot analysis (all PBMTs). The concurrent nuclear accumulation of VDR and the upregulation of CYP24A1 and BGLAP therefore provide a coherent functional readout of ligand-dependent VDR activation, nuclear import, and VDRE-driven transcription. This confirms that 1,25D3 engages the canonical genomic vitamin D signaling pathway in primary malignant bone tumor cells and supports the interpretation that the phenotypic changes observed in viability, apoptosis, and migration arise downstream of bona fide VDR-mediated transcriptional programs.

Additionally, all tumor cell lines expressed CYP27B1, indicating the ability to synthesize active vitamin D endogenously. The regulation of extrarenal CYP27B1, which is necessary for this process, differs from that of the renal variant. The organism strictly regulates the classical renal synthesis pathway of vitamin D hormone, maintaining tight control over 1,25D3 serum levels compared to the storage form 25(OH)D. This regulation spans from a complex endocytosis process into renal cells to hormonal and other regulatory factors such as parathyroid hormone, fibroblast growth factor 23, calcium, and phosphate levels [27], [28]. In peripheral tissues, this conversion step is subject to different regulatory mechanisms, which are influenced primarily by the substrate supply and local factors such as cytokines and growth factors [29]. Extrarenally produced 1,25D3 is commonly linked to nonclassical, extraskeletal effects [30], [31]. CYP27B1 expression in all four cell lines suggests a potential intrinsic increase in cell differentiation, proliferation, and apoptosis upon 25D3 administration. In addition to this canonical pathway, the mitochondrial enzyme CYP11A1 can metabolize vitamin D3 via a non-canonical route to produce biologically active hydroxyderivatives such as 20D3, 22D3, and 20,23D3. These metabolites act independently of the VDR, instead signaling through the AhR, as well as RORα and RORγ. They accumulate in the epidermis, where they exert antiproliferative and antitumoral effects while protecting keratinocytes from UVB-induced damage [9]. However, it cannot be excluded that these hydroxyvitamin D3 metabolites exert antitumoral effects on PMBTs.

Notably, variations in the expression of the catabolic enzyme CYP24A1 were observed across different cell lines. Cell lines derived from chondrosarcoma (SW-1353) and Ewing's sarcoma (RD-ES) demonstrated the ability to produce 1,25D3 but lacked the capacity for its deactivation. CYP24A1, a mitochondrial inner-membrane cytochrome P450 enzyme, plays a central role in catabolic breakdown, catalyzing both the C24-oxidation and the C23-hydroxylation pathways [8]. The absence of this enzyme leads to prolonged half-lives of 1,25D3 in serum and tissues [8], [32]. Studies on *Cyp24a1*-null mice revealed a 20-fold extension in the serum half-life of 1,25D3 compared with that of heterozygous *Cyp24a1*+/− controls [33]. Intriguingly, in certain tumor types, elevated CYP24A1 expression has been observed, contributing to increased 1,25D3 deactivation [13], [34]. In Ewing's and chondrosarcoma cell lines, the absence of CYP24A1 expression upon 1,25D3 treatment is a striking finding with potential translational significance. Unlike osteosarcoma cells, which rapidly degrade active vitamin D via CYP24A1 induction, these two sarcoma types lack this catabolic enzyme and may therefore maintain higher local concentrations of 1,25D3 for extended periods. This could prolong receptor engagement and downstream signaling; however, in our models this did not translate into clear effects on viability in RD-ES cells and only modestly reduced viability in SW-1353 cells (see below). Thus, while altered vitamin D metabolism may influence cellular responses, its functional consequences appear to be context- and cell type-dependent. These findings suggest that bone sarcoma subtypes lacking CYP24A1 induction may still represent a biologically distinct group, warranting further investigation into 1,25D3 responsiveness and patient stratification based on CYP24A1 expression profiles.

After a cell line-specific assessment of reactivity and enzyme profiles, various effects of 1,25D3 on cell physiology were identified. Notably, a consistent reduction in viability was observed in MG-63 osteosarcoma cells across all tested concentrations. This decrease (18–23%) contrasts with the slight upregulation of viability detected in SaOS-2 cells, highlighting cell line-dependent responses even within the same tumor subtype. Furthermore, SW-1353 chondrosarcoma cells exhibited a modest but significant reduction in viability when exposed to 10^−7^ M 1,25D3, indicating that the impact of 1,25D3 extends beyond osteosarcoma-derived models. In a study conducted several decades ago, 1,25D3 led to decreased growth in human MG-63 cells, in contrast with the accelerated growth observed in the VDR-expressing rat osteosarcoma cell line ROS 17/2.8 [35]. Our experiments revealed a similar disparity between the two human osteosarcoma cell lines. The partially opposing effects remain largely unclear and necessitate further scientific research. These differential responses may be explained by the distinct biological nature of MG-63 and SaOS-2. MG-63 are more proliferation-oriented, less differentiated, and display an “earlier” osteoblast-like phenotype, making them particularly sensitive to growth- and differentiation-regulating signals such as the 1,25D3/VDR pathway, which can translate into suppressed proliferation and a shift toward osteogenic differentiation. In contrast, SaOS-2 represent a more advanced, mineralization-competent osteoblast phenotype, in which proliferation is naturally lower and matrix formation and differentiation dominate [20]. Accordingly, they appear to interpret 1,25D3 more as a differentiation-supportive stimulus and less as a strong anti-proliferative signal. The partially opposing effects remain largely unclear and necessitate further scientific research.

The viability data were further supported by cell-cycle analyses, which revealed a cell line-dependent response to 1,25D3. In particular, the observed redistribution of MG-63 cells toward the G1 phase is consistent with a cytostatic effect of vitamin D signaling and provides a plausible explanation for the reduced viability detected in this cell line. By contrast, the limited or absent changes in cell-cycle progression in SaOS-2, SW-1353, and RD-ES cells parallel the comparatively weak effects of 1,25D3 on viability. These findings suggest that the antiproliferative action of 1,25D3 in primary malignant bone tumor cells depends on the cellular context and may primarily involve modulation of cell-cycle progression rather than induction of cell death.

Cell-cycle arrest in the G0/G1 phase has been reported previously as a hallmark of 1,25D3-mediated growth control in MG-63 osteosarcoma cells [36] and various malignancies [37] and is frequently associated with enhanced cellular differentiation and suppression of proliferative signaling pathways [36], [37]. The present findings are consistent with this concept and support the notion that the biological effects of 1,25D3 in osteosarcoma cells are predominantly cytostatic. This interpretation is further strengthened by the concomitant induction of osteogenic markers and the absence of increased apoptosis following treatment.

Furthermore, 1,25D3 consistently reduced apoptosis rates by at least 18–25% across all concentrations in both osteosarcoma cell lines, whereas chondro- and Ewing's sarcoma cells remained unaffected. Vitamin D is commonly associated with an apoptosis-inducing effect on tumor cells, as observed in colon and prostate carcinoma cells [38], [39]. However, the actual data landscape is complex, suggesting that active vitamin D can both initiate and suppress programmed cell death. Our data align with those of a cross-species study on the effects of 1,25D3 on osteosarcoma cells. Analysis of human MG-63 cells via Annexin-V staining revealed reduced rates of apoptosis and growth inhibition [40]. This pattern was also observed in acute myeloid leukemia cells [41]. The reduction in apoptosis might be associated with the initiation of the monocytic differentiation pathway. Similar behavior was observed in osteoblasts and melanocytes, where 1,25D3 led to enhanced differentiation and reduced programmed cell death [42], [43]. This differentiation-driven shift toward a more mature phenotype may help explain the reduced apoptosis in both osteosarcoma cell lines. In MG-63 and SaOS-2, 1,25D3 promotes osteogenic differentiation, as evidenced by increased expression of the vitamin D-responsive osteogenic marker BGLAP, which likely favors a quiescent, matrix-stabilizing state over a proliferative, apoptosis-prone one. Rather than acting primarily as a pro-apoptotic agent in this context, 1,25D3 appears to redirect cells toward differentiation-associated survival, thereby lowering the spontaneous apoptotic fraction despite overall growth inhibition. In summary, vitamin D hormone does not seem to exert its growth-inhibiting effects on primary malignant bone tumor cells through the initiation of apoptosis. Deeper insights might be gained through real-time assays and characterization of the differentiation process triggered by 1,25D3.

The migration of tumor cells, together with the degradation of the surrounding matrix, is a crucial part of the initial metastatic process [44]. We therefore assessed the cell's migration via a wound healing assay. Live-cell imaging combined with AI-based quantification of the cell-covered growth area revealed up to a 15% reduction in migration of MG-63 following 1,25D3 treatment. In contrast, no consistent or statistically significant effect of 1,25D3 on SaOS-2, SW-1353, or RD-ES cell migration was observed, although a trend toward reduced migration was apparent. Migration inhibition upon 1,25D3 administration has been reported previously in breast, thyroid, and prostate carcinoma cells [45], [46], [47]. Importantly, commonly used migration assays have an inherent limitation in distinguishing migration from proliferation, and this limitation also applies to the data presented in this manuscript. While our study establishes that 1,25D3 treatment affects migration/proliferation in a cell line-dependent manner, the precise molecular mechanisms remain to be elucidated. Potential confounding factors include cytostatic effects (reduced proliferation) and differentiation-induced changes in cell motility. A comprehensive transcriptomic analysis via RNA-seq across the different bone sarcoma entities would be highly impactful to identify 1,25D3-regulated pathways governing migration, adhesion, and cytoskeletal organization, followed by functional validation of key genes. These extensive analyses are beyond the scope of the present study but represent critical next steps.

In conclusion, our findings demonstrate that 1,25D3 affects the viability, apoptosis, and migration of primary malignant bone tumor cells. Treatment with 1,25D3 reduced viability in MG-63 osteosarcoma cells and decreased the apoptosis rate in both osteosarcoma lines (MG-63 and SaOS-2). In contrast, the SW-1353 chondrosarcoma and RD-ES Ewing's sarcoma cells remained largely unaffected. Moreover, 1,25D3 suppressed cell migration in MG-63 cells, with a similar, though less pronounced, inhibitory trend observed in SaOS-2, SW-1353, and RD-ES cells. Distinct gene expression patterns further highlight tumor-specific responses to 1,25D3. Notably, the catabolic enzyme CYP24A1 was expressed in osteosarcoma lines (MG-63 and SaOS-2) but absent in Ewing's sarcoma and chondrosarcoma lines, suggesting differences in their capacity to degrade 1,25D3. Future research should concentrate on the conditions under which the variable effects are induced, providing further insights into when potential vitamin D supplementation could be beneficial for patients affected by primary malignant bone tumors.

## CRediT authorship contribution statement

**M. Hein:** Writing – review & editing, Writing – original draft, Visualization, Methodology, Investigation, Formal analysis. **S. Zeck:** Visualization, Validation, Methodology, Formal analysis. **M. Krug:** Visualization, Validation, Formal analysis. **J. Meißner-Weigl:** Validation, Methodology. **O. Bahr:** Formal analysis, Methodology, Writing – review & editing. **K. Kriukov:** Visualization, Formal analysis. **M. Kuric:** Visualization, Methodology. **D. Docheva:** Writing – review & editing, Funding acquisition. **M. Rudert:** Writing – review & editing. **K. Horas:** Writing – review & editing, Writing – original draft, Formal analysis, Conceptualization. **R. Ebert:** Writing – review & editing, Writing – original draft, Visualization, Validation, Supervision, Project administration, Methodology, Funding acquisition, Formal analysis, Conceptualization.

## Funding

This work was funded by the 10.13039/501100001659Deutsche Forschungsgemeinschaft (DFG, German Research Foundation) - Project number 491715122 – microBone (R.E.).

## Declaration of competing interest

The authors declare that they have no known competing financial interests or personal relationships that could have appeared to influence the work reported in this paper.
