# Supplementary material for: Vitamin D decreases tumor cell growth and invasiveness in primary malignant bone tumors in vitro
Source: J Bone Oncol. 2026 Jul 31;60:100792. doi: 10.1016/j.jbo.2026.100792 (PMC13470584; doi:10.1016/j.jbo.2026.100792)
Supplement: Supplementary file 1 [file mmc1.pdf]

Supplementary Information

Vitamin D decreases tumor cell growth and invasiveness in primary malignant bone tumors in vitro

Hein M.[a], Zeck S.[a], Krug M.[a], Meißner-Weigl J.[a], Bahr O.[a], Kriukov K.[a], Kuric M.[a], Docheva D.[a], Rudert M.[b], Horas K.[b,c,#], Ebert R.[a,#,*]

[1]University of Würzburg, Department of Musculoskeletal Tissue Regeneration, Orthopedic Hospital König-Ludwig-Haus, Friedrich-Bergius-Ring 15, 97076 Würzburg, Germany

[2]University of Würzburg, Department of Orthopedics, Orthopedic Hospital König-Ludwig-Haus, Brettreichstrasse 11, 97074 Würzburg, Germany

[3]ATOS Klinik Wiesbaden, Hagenauer Straße 47, 65203 Wiesbaden, Germany

*: corresponding author:

Regina Ebert

regina.ebert@uni-wuerzburg.de

[#]: R. Ebert and K. Horas contributed equally.

Basal VDR expression

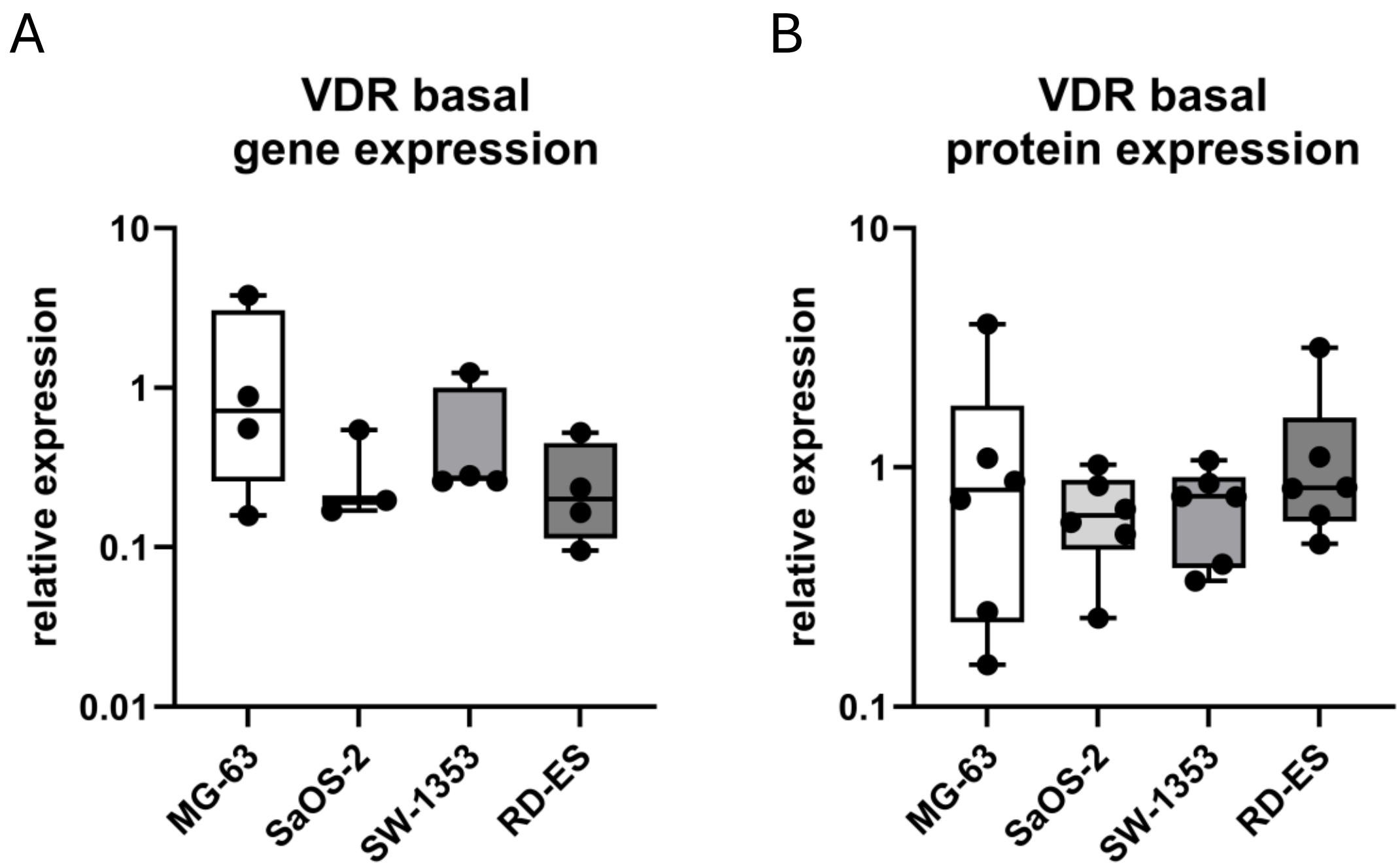

Fig. S1: Basal expression of VDR. Basal mRNA expression of VDR was analyzed by RT-PCR in MG-63, SaOS-2, SW-1353, and RD-ES cells **(A)**. Band densities were quantified using ImageJ and normalized to the housekeeping gene RPS27A. Basal VDR protein expression in nuclear extracts was analyzed by Western blot in MG-63, SaOS-2, SW-1353, and RD-ES cells **(B)**. Band densities were quantified using ImageJ and normalized to the loading control Lamin B1. Data are presented as boxplots from four (mRNA) or six (nuclear extracts) independent experiments, with individual data points shown. Statistical analysis was performed using one-way ANOVA in GraphPad Prism; no significant differences were detected. Abbreviations: RPS27A, ribosomal protein S27a; VDR, vitamin D receptor.

1,25D3 effects on gene expression

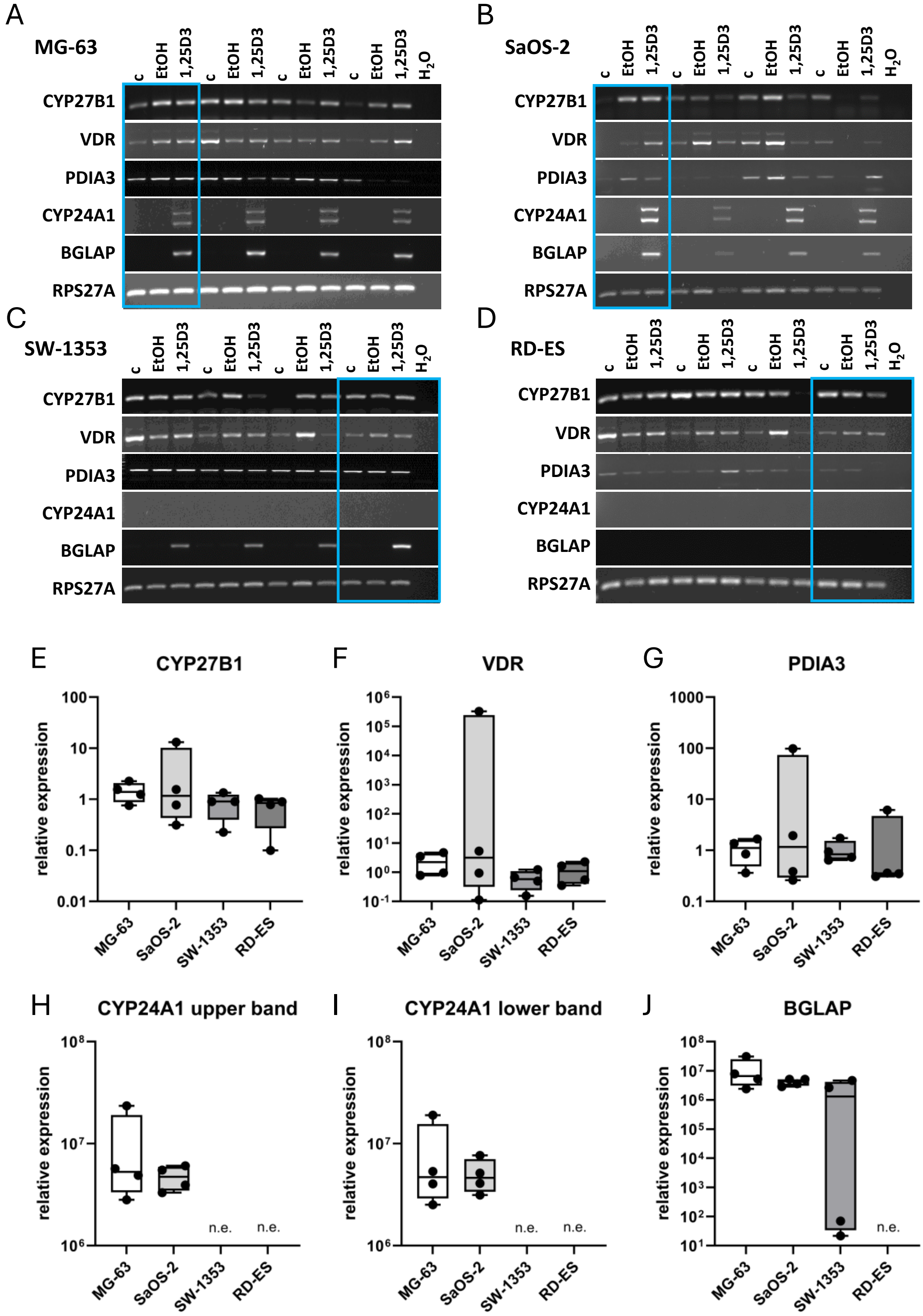

Fig. S2: 1,25D3 effects on gene expression. MG-63 **(A)**, SaOS-2 **(B)**, SW-1353 **(C)**, and RD-ES **(D)** cells were stimulated with 10^{-7} M 1,25D3, with 0.01% ethanol as the solvent control (EtOH), for 24 h. Untreated control samples are labeled (c). The expression of CYP27B1, VDR, PDIA3, CYP24A1, BGLAP, and RPS27A as the housekeeping gene was analyzed via RT-

PCR. Four independent experiments are shown, the blue box marks the bands that are also shown in the main manuscript as Fig. 1. Quantification of CYP27A **(E)**, VDR **(F)**, PDIA3 **(G)**, CYP24A1 upper band (transcript variant 5) **(H)**, CYP24A1 lower band (transcript variant 1) **(I)**, and BGLAP **(J)** band densities measured using ImageJ and normalized to the housekeeping gene RPS27A. Data are presented as boxplots from four independent experiments, with individual data points shown. Abbreviations: BGLAP, bone gamma-carboxyglutamate protein; CYP24A1, 24-hydroxylase, CYP27B1, 1α-hydroxylase; n.e., not expressed; PDIA3, protein disulfide isomerase A3; RPS27A, ribosomal protein S27a; VDR, vitamin D receptor.

Immunocytochemical staining of the secondary antibody as negative control

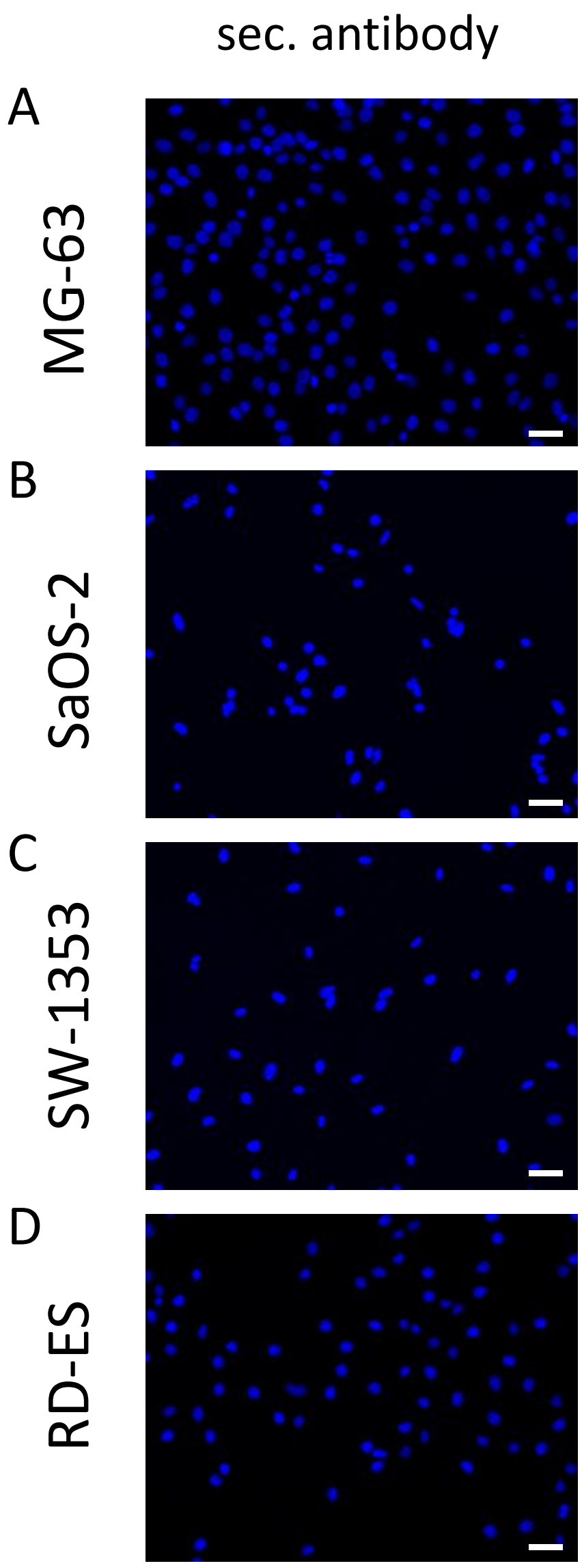

Fig S3: Immunocytochemical staining of the secondary antibody as negative control. MG-63 (**A**), SaOS-2 (**B**), SW-1353 (**C**) and RD-ES (**D**) cells treated with 10^{-7} M 1,25D3 for 2 h. Alexa Fluor 488-conjugated secondary antibody was used, no staining could be detected. Blue staining = DAPI. Bar represents 50 µm. Representative images of two or three independent experiments are shown.

Wound healing assays, experiment 2 and 3

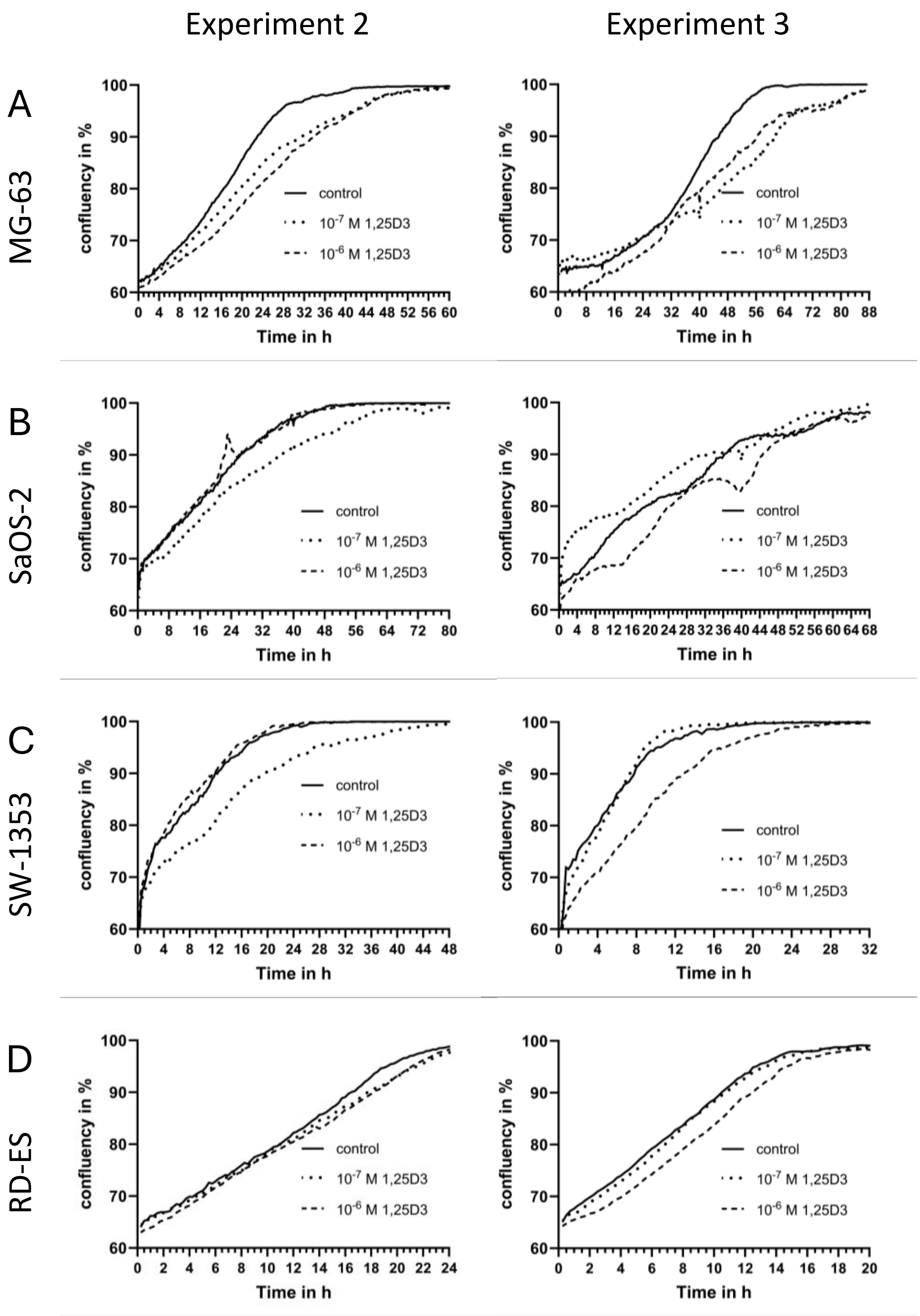

Fig. S4: Wound healing assays, experiment 2 and 3. (A) MG-63, (B) SaOS-2; (C) SW-1353, and (D) RD-ES cells were exposed to 10^{-6} M and 10^{-7} M 1,25D3 and to 0.01% ethanol (solvent control), quantification of the gap closure for indicated time points.

		24 h			48 h		
	cell cycle phase	mean difference in % (1,25D3 vs. EtOH)	significance	p-value	mean difference in % (1,25D3 vs. EtOH)	significance	p-value
MG-63	G1	11.78	***	0.0005	4.487	**	0.0032
	G2	-6.38	*	0.0384	-2.91	*	0.0495
	S	-5.4	ns	0.0864	-1.243	ns	0.5923
SaOS-2	G1	-0,436	ns	0.9939	2,487	ns	0.7206
	G2	-0,1723	ns	0.9996	0,68	ns	0.9912
	S	0,6111	ns	0.9838	-3,167	ns	0.5535
SW-1353	G1	1.853	ns	0.5859	-2.37	ns	0.1584
	G2	0.0367	ns	>0.9999	-0.0033	ns	>0.9999
	S	-1.883	ns	0.5738	2.293	ns	0.1776
RD-ES	G1	-3.777	ns	0.7593	-2.443	ns	0.7674
	G2	-4.613	ns	0.6349	-5.75	ns	0.157
	S	8.387	ns	0.182	8.193	*	0.0314

Tab. S1: Effects of 1,25D3 on G1, S, and G2 cell-cycle phase distribution in MG-63, SaOS-2, SW-1353, and RD-ES cells. Mean differences (%) between 1,25D3-treated controls and EtOH-treated cells after 24 h and 48 h are shown. Statistical significance was assessed by two-way ANOVA using GraphPad Prism, followed by Šídák's multiple-comparisons test for post hoc pairwise comparisons; significance levels and corresponding p-values are indicated. Three independent experiments were performed and analyzed.
